# A Meta-analysis to Quantify the Risk of Disseminated Gonococcal Infection With Porin B Serotype

**DOI:** 10.1093/ofid/ofae389

**Published:** 2024-07-08

**Authors:** Geoffrey Welch, George W Reed, Peter A Rice, Sanjay Ram

**Affiliations:** Division of Infectious Diseases and Immunology, Department of Medicine, University of Massachusetts Chan Medical School, Worcester, Massachusetts, USA; Division of Infectious Diseases and Immunology, Department of Medicine, University of Massachusetts Chan Medical School, Worcester, Massachusetts, USA; Division of Infectious Diseases and Immunology, Department of Medicine, University of Massachusetts Chan Medical School, Worcester, Massachusetts, USA; Division of Infectious Diseases and Immunology, Department of Medicine, University of Massachusetts Chan Medical School, Worcester, Massachusetts, USA

**Keywords:** disseminated gonococcal infection, gonorrhea, *Neisseria gonorrhoeae*, PorB, serotype

## Abstract

The escalating rates of gonorrhea globally are associated with higher numbers of disseminated gonococcal infection (DGI). Expression of the PorB1A allele of the major outer membrane porin protein, PorB, is associated with DGI. This meta-analysis shows that the odds of PorB1A strains to disseminate is 20.53 compared to PorB1B isolates.

*Neisseria gonorrhoeae* (*Ng*) is the causative organism of the sexually transmitted infection gonorrhea. The World Health Organization estimated a total of 82.4 million new cases of gonorrhea in 2020 [1]. Uncommonly, gonococcal infections can lead to disseminated disease that manifests as dermatitis, arthritis, and, rarely, endocarditis or meningitis. The climbing rates of gonorrhea globally have been associated with increased reports of disseminated gonococcal infection (DGI) [2–5].

The traditionally cited rate of DGI is 0.5%–3% of all cases of gonorrhea [6–11]. Host and microbial factors both contribute to the risk of developing DGI. About two-thirds of DGI cases occur in women; menstruation is a risk factor and symptoms happen within 7 days of menses in half of affected women. Congenital deficiencies of complement, especially the terminal complement components (C5–C9) that are involved in the assembly of bactericidal membrane attack complex, predispose to DGI [12–16]. Pharmacologic blockade of C5 with eculizumab also predisposes to DGI [17]. Porin B (PorB; previously called Protein I) is the most abundant outer membrane *Ng* protein [18] and may play an important role in gonococcal dissemination. *Ng* expresses 1 of 2 PorB alleles (either PorB1A or PorB1B), and *Ng* is often categorized (or grouped) based on the type of PorB expressed [19]. The majority of disseminated infections are caused by PorB1A-expressing isolates [2, 7, 20, 21]. To quantitate the likelihood, or odds, of PorB1A isolates to disseminate compared to PorB1B strains, we performed a meta-analysis.

## METHODS

We reviewed the available literature on PubMed using the search term “disseminated gonococcal infection” to identify case series of gonorrhea that also characterized strains based on PorB serovar or their *porB* gene and separated strains into PorB1A versus PorB1B types. Further, each included study also linked PorB-type clinical presentation—local (uncomplicated) versus disseminated infection. Inclusion criteria were determined following the Population, Exposure, Comparison, Outcome (PECO) model. The population included persons within a defined geographic region (such as a city, state, or country) in whom *Ng* was detected by either nucleic acid amplification test or culture and the PorB was typed either with monoclonal antibodies or by DNA sequencing over a defined time period. The exposure and comparison groups were defined as the presence of the PorB1A or PorB1B pheno/genotype in the *Ng* isolates, respectively. The outcome was the proportion of localized gonococcal infection versus DGI in exposure versus comparison group. Studies that contained only localized infection without clinical evidence of dissemination, disseminated infection only, or that did not differentiate between the PorB phenotypes were excluded.

A 2-stage process meta-analysis was carried out using the “meta” programs esize, forestplot, and funnelplot in Stata version 18.0 software (StataCorp LLC, College Station, Texas). It involves the estimation of an appropriate summary statistic—odds ratios (ORs)—for each of a set of studies followed by the calculation of a weighted average of these statistics across the studies [22]. The summary statistics used a random-effects model. The Q statistic was used to test for homogeneity of odds. Three measures of heterogeneity of effect sizes were estimated—T^2^ (tau), I^2^, and H^2^ [23–25]. A funnel plot was generated to examine any potential bias [26].

## RESULTS

The systematic search returned a total of 587 citations with zero duplicates. After full-text review, only 6 studies met the PECO criteria and were included for analysis (Bohnhoff et al, 1986 [27], Guglielmino et al, 2022 [28], Cartee et al, 2022 [2], Sandstrom et al, 1984 [21], Tapsall et al, 1992 [29], and Brunham et al, 2022 [30]). The selected studies were all retrospective surveillance studies and represented populations in Australia, Manitoba (Canada), and the following major cities in the United States: Chicago, Atlanta, Denver, and Seattle. Each study looked at individual strains of *Ng* collected from patients over a defined time period. The mean data collection time was 3.9 years, ranging from 1 year to 11 years. Each study reported measures taken to exclude reporting of duplicate isolates from the same individual, so that each reported isolate correlated to a single clinical case. With a single exception (Bohnhoff et al [27]), DGI cases in all studies were defined by isolation of *Ng* from a normally sterile body site, such as blood, joint fluid, skin lesions, spinal fluid, or peritoneal fluid (Supplementary Table 1). The 137 DGI cases reported by Bohnhoff et al [27] included 50 patients with characteristic signs and/or symptoms of DGI, where *Ng* was not recoverable from a sterile site but only from mucosal surfaces (suspected DGI); these suspected DGI cases were included in our analysis.

Across all 6 studies, a total of 6537 isolates of *Ng* were collected. Of these isolates, 1929 expressed the *porB1a* gene (29.5%). A total of 358 cases of DGI were documented (5.5%); 310 of these cases of DGI were caused by isolates that expressed the *porB1A* gene (86.6%).

**Figure 1.**
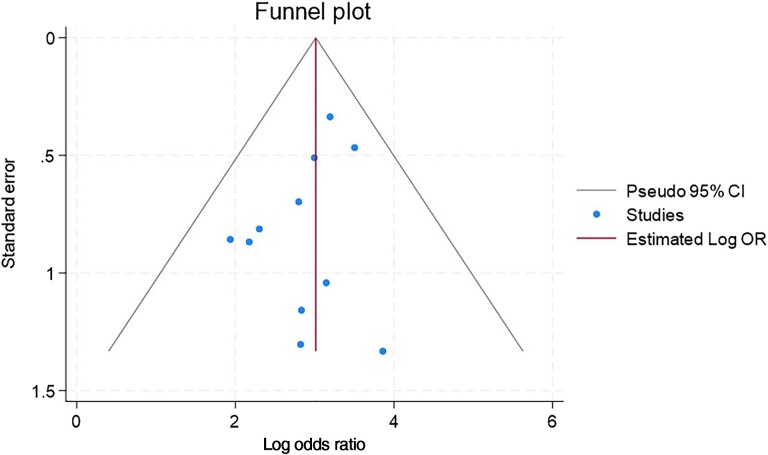
A funnel plot was generated using the log odds ratio (OR) from each study plotted against the standard error from each study. The 95% confidence interval (CI) is shown as the gray outline. All studies fall within the funnel (estimate ±1.96 standard error), which suggests that the “true treatment effect” is the same in each study and is not biased in underlying populations or study design.

In each individual study, the presence of *porB1a* allele was associated with a statistically significant increase in incidence of disseminated disease. An OR was calculated for each study. The calculated ORs ranged from 6.93 to 55.54. Statistical weight was assigned to each study based on sample size. The overall OR based on statistical weight was 20.53 (Table 1). The log OR of each study was shown on a funnel plot, and all studies were within the predicted confidence interval (Figure 1). The tests of heterogeneity all indicated that variation in OR estimates are due to sampling error—evidence that the true underlying OR is similar in the studies.

**Table 1.** Meta-analysis to Determine the Odds Ratio of PorB1A *Neisseria gonorrhoeae* to Cause Disseminated Gonococcal Infection

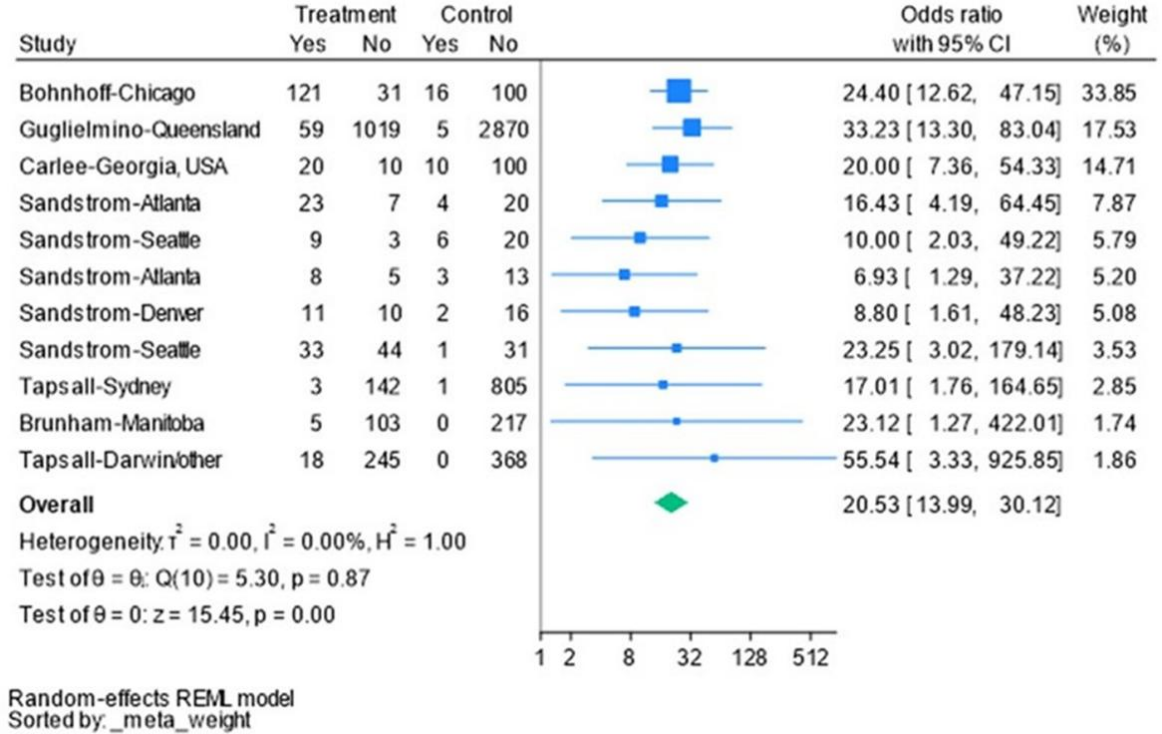

I^2^ can be interpreted as the percentage of the total variability in a set of effect sizes due to true heterogeneity, that is, to between-studies variability. For example, a meta-analysis with I^2^ = 0 means that all variability in effect size estimates is due to sampling error within studies. τ^2^ provides a measure of between study variance based on random-effects model. The H statistic is interpreted as the ratio of the standard deviation of the estimated overall effect size from a random-effects meta-analysis compared to the standard deviation from a fixed-effect meta-analysis.

Abbreviation: CI, confidence interval.

## DISCUSSION

Several properties of PorB1A strains may enable them to disseminate more readily than their PorB1B counterparts. Under low-phosphate conditions, as occurs in plasma, PorB1A strains traverse epithelial cells more readily by virtue of PorB1A interactions with scavenger receptor on endothelial cells 1 and human heat shock glycoprotein 86 (gp86) [31, 32]. Evasion of complement and survival in the bloodstream is a prerequisite for dissemination. About 90% of PorB1A isolates bind the classical pathway inhibitor C4b-binding protein (C4BP) [33, 34]. A majority of DGI-causing strains also bind to Factor H (FH) [35], an inhibitor of the alternative pathway of complement. C4BP and FH bound to the gonococcal surface limit the amount of C4b and C3b, respectively, deposited on the bacterial surface and thus prevent formation of lytic membrane attack complex (MAC, or C5b-9). Inhibition of complement activation also limits the generation of C5a, a potent anaphylatoxin and chemoattractant for neutrophils [36]. Accordingly, a paucity of local genital inflammatory signs and symptoms is a hallmark of DGI [6, 7]. Asymptomatic gonococcal infections occur in about 20% of men and 50% of women [37]. Whether PorB1A isolates are associated with asymptomatic infections more frequently is unclear and merits study.

Here, we show that PorB1A strains are about 20 times more likely to disseminate relative to PorB1B isolates. Two recent studies that interrogated large whole genome databases have shown that PorB1A isolates are a minority and comprise only about 9.4% of all *Ng* isolates [38, 39]. The PorB1B preponderance among non-DGI isolates is also evident in the studies included in our analysis (Figure 1). The particularly invasive clone of *Ng* (the arginine/hypoxanthine/uracil-requiring [AHU^–^] auxotype that later was typed as a Por1A-1 serovar) that was responsible for 89% of DGI cases (and a rate of DGI approaching 3%) during 1973–1974 was responsible for 38% of cases of uncomplicated gonorrhea [40]. Studies in Australia in the late 1980s cited rates of DGI of 0.08% in Sydney and 0.87% in Darwin, where the prevalence of PorB1A strains was 15% and 40%, respectively [29]. These data suggest that differences in the rates of DGI may, in large part, be driven by the prevalence of circulating PorB1A isolates. Therefore, we believe that ongoing surveillance and characterization of gonococcal isolates is important because an increase in circulating PorB1A isolates could portend an increase in DGI. For reasons not entirely clear, the distribution of gonococcal clones (or serovars) fluctuates over time in populations. The AHU^–^ auxotype constituted 46 of 84 (55%) strains in Lübeck, Germany, from 1976 to 1978, but only 17 of 94 (18%) isolates from 1980 to 1982 [41].

Our study has several limitations. We acknowledge that there is considerable heterogeneity among PorB1A strains and that certain PorB1A strains may be more likely to disseminate than others. Based on the available data, we were unable to define the subsets (or serovars) of PorB1A strains that are most likely to disseminate. Given that PorB1B strains can also cause DGI [2, 7], we acknowledge that factors other than PorB1A may determine the ability of a strain to disseminate. Similar to most PorB1A strains, about 20%–30% of PorB1B strains bind C4BP and are resistant to killing by complement [33, 34], which could contribute to their potential to disseminate. While we have examined the association between DGI and PorB type, other gonococcal molecules and host factors likely contribute to dissemination and merit further study.

## Supplementary Material

ofae389_Supplementary_Data

## Supplementary Data

Supplementary materials are available at *Open Forum Infectious Diseases* online. Consisting of data provided by the authors to benefit the reader, the posted materials are not copyedited and are the sole responsibility of the authors, so questions or comments should be addressed to the corresponding author.

## Notes

***Patient consent*.** No primary data were used; thus, patient consent was not required. Ethical committee approvals were not required for conducting this meta-analysis.

***Financial support.*** S. R. and P. A. R. are funded by the National Institutes of Health/National Institute of Allergy and Infectious Diseases (grant number R01 AI141181).
