# Supplementary material for: A Meta-analysis to Quantify the Risk of Disseminated Gonococcal Infection With Porin B Serotype
Source: Open Forum Infect Dis. 2024 Jul 8;11(7):ofae389. doi: 10.1093/ofid/ofae389 (PMC11259189; doi:10.1093/ofid/ofae389)
Supplement: ofae389_Supplementary_Data [file ofae389_supplementary_data.docx]

**Supplemental Table 1.** Definition of DGI and uncomplicated infection in studies included in analysis

| **Study [Ref.]** | **Definition/selection of DGI cases** | **Definition/selection of uncomplicated Ng cases** |
| --- | --- | --- |
| Bohnhoff et al [1] | Specimens were obtained with patients with signs/symptoms of DGI. Women with a positive culture and recent abortion or other genitourinary tract manipulation were excluded. For 86 patients, gonococci were isolated from one or more of the following: blood, joint fluid skin lesion, or spinal fluid. The other 50 patients had suspected DGI (signs / symptoms of DGI – i.e., septic arthritis, dermatitis, tenosynovitis syndrome), but isolates were obtained only from the urethra, cervix, oropharynx, or rectum or from a combination of these sites. | Each DGI or suspected DGI patient was matched by sex and date of presentation with a control patient seen in the emergency department with uncomplicated, localized gonorrhea. |
| Guglielmino et al [2] | DGI was defined where the organism was isolated from blood culture, joint fluid and/or tissue (breakdown of numbers from each site not provided). | Non-DGI isolates were from genital (n=3099), anorectal (n=456),  oropharyngeal (n=233), ocular (n=31) and other/not specified (n=70) sites. Symptomatology not documented. |
| Cartee et al [3] | DGI isolates were all derived from normally sterile body sites – blood (n=21), synovial fluid (n=8), or peritoneal fluid (n=1) | Uncomplicated genital infection isolates were collected in the Atlanta area as part of the Gonococcal Isolate Surveillance Project (GISP) and/or Strengthening the US Response to Resistant Gonorrhea (SURRG) program. GISP -  Gonococcal urethral isolates from symptomatic men. SURRG includes specimens from patients of all genders and from genital and  extragenital sites of infection. The number of GISP vs SURRG isolates, or the proportion of isolates from symptomatic persons was not specified. |
| Sandstrom et al [4] | DGI isolates were from normally sterile body sites – blood, joints or skin lesions. | Isolates from patients with uncomplicated gonorrhea – patient symptoms not mentioned.  Seattle 1971-1973: 75 uncomplicated case isolates from participants in gonorrhea therapy trial, not randomly selected or matched with DGI cases.  Seattle 1980-1981: uncomplicated cases matched with DGI cases with respect to age, sex, race and sex preference during the same time period.  Denver 1977-1980: 26 genital isolates selected from each of two patients with uncomplicated infection treated immediately after each of the 13 DGI patient at the same hospital.  Atlanta 1976-1979: DGI isolates from 1976-1979 matched by age, race, sex and sex preference with uncomplicated infection isolates from 1975.  Atlanta 1981-1982: DGI isolates rom 1981 matched by age, race, sex and sex preference with uncomplicated infection isolates from 1982. |
| Tapsall et al [5] | DGI isolates were recovered from blood or joint fluid. | 947 isolates from Sydney and 613 isolates cultured from mucosal sites. Symptomatology of patients not mentioned. |
| Brunham et al [6] | Typical cutaneous lesions, tenosynovitis, and/or arthritis together with isolation of *N. gonorrhoeae* from blood, joint fluid, or mucosal surface constituted the basis for diagnosis of DGI. Two strains isolated from blood and one from synovial fluid. | Isolates obtained from symptomatic and asymptomatic individuals. Isolates were from the urethra (n=168), cervix (n=137), anal canal (n=6), pharynx (n=7), conjunctival sac (n=1) and Bartholin’s abscess (n=1). Of the urethritis isolates, 58 were PorB1A (3 were asymptomatic) and 110 were PorB1B (12 were asymptomatic). |

**References**

1. Bohnhoff M, Morello JA, Lerner SA. Auxotypes, penicillin susceptibility, and serogroups of Neisseria gonorrhoeae from disseminated and uncomplicated infections. J Infect Dis **1986**; 154:225-30.

2. Guglielmino CJD, Sandhu S, Lau CL, et al. Molecular characterisation of Neisseria gonorrhoeae associated with disseminated gonococcal infections in Queensland, Australia: a retrospective surveillance study. BMJ Open **2022**; 12:e061040.

3. Cartee JC, Joseph SJ, Weston E, et al. Phylogenomic Comparison of Neisseria gonorrhoeae Causing Disseminated Gonococcal Infections and Uncomplicated Gonorrhea in Georgia, United States. Open Forum Infect Dis **2022**; 9:ofac247.

4. Sandstrom EG, Knapp JS, Reller LB, Thompson SE, Hook EW, 3rd, Holmes KK. Serogrouping of Neisseria gonorrhoeae: correlation of serogroup with disseminated gonococcal infection. Sex Transm Dis **1984**; 11:77-80.

5. Tapsall JW, Phillips EA, Shultz TR, Way B, Withnall K. Strain characteristics and antibiotic susceptibility of isolates of Neisseria gonorrhoeae causing disseminated gonococcal infection in Australia. Members of the Australian Gonococcal Surveillance Programme. Int J STD AIDS **1992**; 3:273-7.

6. Brunham RC, Plummer F, Slaney L, Rand F, DeWitt W. Correlation of auxotype and protein I type with expression of disease due to Neisseria gonorrhoeae. J Infect Dis **1985**; 152:339-43.
